# Deoxyribonucleic Acid Damage and Repair: Capitalizing on Our Understanding of the Mechanisms of Maintaining Genomic Integrity for Therapeutic Purposes

**DOI:** 10.3390/ijms19041148

**Published:** 2018-04-11

**Authors:** Jolene Michelle Helena, Anna Margaretha Joubert, Simone Grobbelaar, Elsie Magdalena Nolte, Marcel Nel, Michael Sean Pepper, Magdalena Coetzee, Anne Elisabeth Mercier

**Affiliations:** 1 Department of Physiology, School of Medicine, Faculty of Health Sciences, University of Pretoria, Pretoria 0002, South Africa; jolenehelena@gmail.com (J.M.H.); annie.joubert@up.ac.za (A.M.J.); simonegrow@gmail.com (S.G.); elsa07.nolte@gmail.com (E.M.N.); marcelverwey4@gmail.com (M.N.); magdalena.coetzee@up.ac.za (M.C.); 2 Institute for Cellular and Molecular Medicine, Department of Immunology, South African Medical Research Council (SAMRC) Extramural Unit for Stem Cell Research and Therapy, Faculty of Health Sciences, University of Pretoria, Pretoria 0002, South Africa; michael.pepper@up.ac.za

**Keywords:** DNA replication, DNA damage, DNA repair, genome integrity

## Abstract

Deoxyribonucleic acid (DNA) is the self-replicating hereditary material that provides a blueprint which, in collaboration with environmental influences, produces a structural and functional phenotype. As DNA coordinates and directs differentiation, growth, survival, and reproduction, it is responsible for life and the continuation of our species. Genome integrity requires the maintenance of DNA stability for the correct preservation of genetic information. This is facilitated by accurate DNA replication and precise DNA repair. DNA damage may arise from a wide range of both endogenous and exogenous sources but may be repaired through highly specific mechanisms. The most common mechanisms include mismatch, base excision, nucleotide excision, and double-strand DNA (dsDNA) break repair. Concurrent with regulation of the cell cycle, these mechanisms are precisely executed to ensure full restoration of damaged DNA. Failure or inaccuracy in DNA repair contributes to genome instability and loss of genetic information which may lead to mutations resulting in disease or loss of life. A detailed understanding of the mechanisms of DNA damage and its repair provides insight into disease pathogeneses and may facilitate diagnosis and the development of targeted therapies.

## 1. Deoxyribonucleic Acid as Hereditary Material

Deoxyribonucleic acid (DNA) is the hereditary material found in humans, other eukaryotes, and prokaryotes that carries instructions for structure and function [1]. Acting as a blueprint in collaboration with environment cues, DNA gives rise to phenotype. Accordingly, its integrity is essential for life [2]. Genomic stability is maintained by the accurate replication and adequate repair of DNA; failure of these crucial processes results in DNA damage and the inability to ensure continuation of a given species [3]. The occurrence of DNA damage is more likely to occur at genomic loci which have increased transcriptional activity [4]. Failure to maintain DNA integrity as a result of inadequate repair leads to mutations inducing structural, biochemical, and/or functional aberrations which are the cause of several diseases [2].

## 2. Cell Growth

The purpose of the cell cycle is to generate two genetically identical daughter cells from a single parent cell [5]. This is achieved by the coordination of cell growth, DNA replication, and cell division [5]. The cell cycle is responsive to a variety of cues and signals: internal cellular cues involving DNA damage, external cellular cues, or molecular signals that contribute to the regulation of its progression [5,6]. Cellular cues include hormones and growth factors such as insulin and insulin-like growth factor, nutrients such as amino acids and glucose, and cellular stressors such as hypoxia and osmotic stress [5,6]. The mammalian target of rapamycin (mTOR) protein kinase acts as an environmental sensor to these cues and promotes critical processes of the cell cycle [6].

The cell cycle is divided into interphase and mitosis [7]. During interphase, cell growth and DNA synthesis occur to prepare the cell for mitosis [7]. Interphase consists of the growth 1/gap 1 (G_1_) phase, the DNA synthesis (S) phase, and the pre-mitotic/gap 2 (G_2_) phase, while mitosis comprises the mitotic (M) phase [7]. In the M phase, mitosis is marked by nuclear division and cytokinesis (cytoplasmic division) [8,9]. In the G_1_ phase, cells are metabolically active and grow continuously [8,9]. DNA synthesis and replication occur during the S phase [8,9]. During the G_2_ phase, cells continue to grow and specific proteins are synthesized in preparation for mitosis [8,9]. The resting (G_0_) phase signifies quiescence in which non-dividing cells exit the cell cycle [8,9].

## 3. Cell Cycle Control and Checkpoints

Cell cycle checkpoints are important regulatory mechanisms through which DNA integrity is maintained [10,11]. They only allow cells with stable DNA to undergo DNA replication in the S phase, and only cells with correctly replicated DNA enter the M phase for cell division [10,11]. Any failure of cell cycle control mechanisms leads to a range of mutations resulting from the replication and preservation of damaged and unrepaired DNA [10,11].

Cell cycle control may be described as a three-step process [12,13]. First, DNA synthesis (S phase) and chromosome segregation (M phase) are qualitatively controlled by phosphorylation of various proteins by specific kinases [12,13]. Second, the activity of cyclin-dependent kinases (CDKs) determines the progression of cells through the cell cycle [12,13]. CDKs stimulate the transition between cell cycle phases via phosphorylation of effector protein substrates [5]. CDKs are activated by cyclins and inhibited by CDK inhibitors (CDKIs) [5]. Third, cell cycle-related regulators including cyclins and CDKIs are quantitatively controlled by ubiquitination, an important post-translational modification [12,13]. Ubiquitination results from an enzymatic cascade that involves the attachment of ubiquitin to a lysine residue of the target protein [14]. Target proteins are defined as polypeptides enriched in proline, glutamic acid, serine, and threonine residues which serve as intramolecular signals for proteolytic degradation [15]. This post-translational modification regulates vital cellular activities such as cell growth and death, chromatin organization and dynamics, gene expression, and the DNA damage response (DDR) [14].

**Figure 1:**
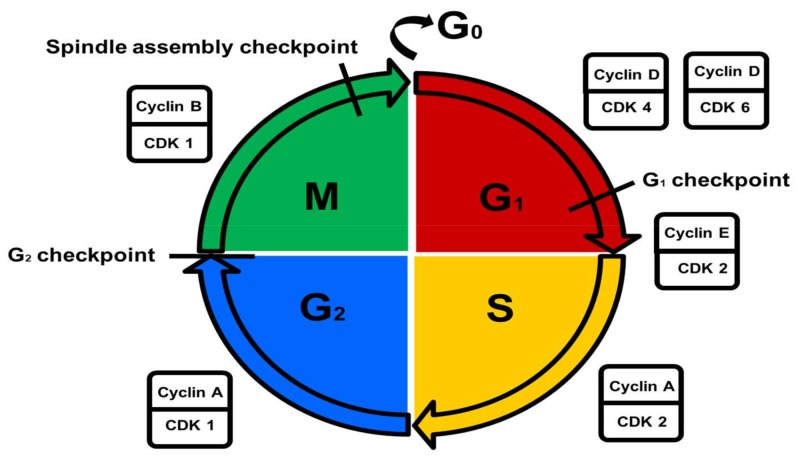
Control of the cell cycle. Metabolically active growing cells are present in the growth 1/gap 1 (G_1_) phase. DNA replication occurs in the DNA synthesis (S) phase. Cells prepare for mitosis in the pre-mitotic/gap 2 (G_2_) phase. Cells undergo nuclear- and cytoplasmic division in the mitotic (M) phase. Non-dividing cells exit the cell cycle in the resting (G_0_) phase. The different cell cycle phases are regulated by specific cyclin/cyclin-dependent kinase (CDK) complexes.

CDK regulation is controlled by the nuclear availability of cyclins throughout the cell cycle, phosphorylation by CDK activating kinases (CAKs), and the activity of CDKI peptide inhibitors [16]. Cyclins are a family of proteins that control the progression of the cell cycle by forming complexes with CDKs thereby modulating CDK activation and activity [16,17]. Cyclin D-CDK4 and -D-CDK6 regulate the G_1_ phase, cyclin E-CDK2 is responsible for the G_1_/S phase transition, cyclin A-CDK2 regulates the S phase, cyclin A-CDK1 regulates the G_2_ phase, and cyclin B-CDK1 is involved in the regulation of the M phase [17]. CAKs activate all CDKs, whereas only a few inhibited by Wee1- and myelin transcription factor 1 (Myt1) kinases and promoted by cell division cycle 25 (cdc25) phosphatases [16]. Active cyclin-CDK complexes are inactivated by the binding of CDKIs from either the CDK4 inhibitor (INK) family (p15, p16, p18 and p19) or the CDK inhibitor (KIP) family (p25, p27 and p57) [16]. The INK family of CDKIs is capable of inhibiting all CDKs, whereas the KIP family of CDKIs can only inhibit CDKs involved in the G_1_ phase (Figure 1) [5].

The G_1_ checkpoint ensures that cell size is adequate, that nutrient supply is sufficient, that growth factors are present, and that there is no DNA damage [10,18]. The G_2_ checkpoint ensures that error-free DNA replication occurs by activating DNA repair mechanisms during an induced pause in the cycle if required [10,18]. Near the end of the M phase, the spindle assembly checkpoint ensures that chromosomes are stably attached to the mitotic spindle to facilitate chromosome separation [10,18].

## 4. Disruption of Genome Integrity

DNA damage is defined as chemical (dynamic) and physical (structural) alterations to the DNA double helix that are derived from endogenous or exogenous origins and impair the function and integrity of DNA [19,20].

If the damaged DNA is repairable, the necessary cell cycle checkpoints are activated, the DNA is repaired, genome integrity is restored, and the cell survives [21]. If the extent of DNA damage is irreparable, cells containing damaged DNA are directed to undergo senescence or programmed cell death to prevent the proliferation of mutant cells and the replication of erroneous DNA [21]. Should DNA repair mechanisms and DNA damage elimination processes fail, mutations and chromosomal aberrations arise which may lead to malignant and pathological transformation of the cell [21,22].

## 5. Endogenous Deoxyribonucleic Acid Damage

Endogenous DNA damage, originating from internal metabolic processes, includes damage caused by reactive oxygen species (ROS) and reactive nitrogen species (RNS) [5,20]. These products are formed during oxidative stress, metabolic processes, and the inflammatory response [5,20]. Endogenous DNA damage also includes depurination and depyrimidination at certain loci [19,20]. This occurs through the hydrolysis of *N*-glycosidic bonds between nitrogenous bases and deoxyribose residues, resulting in apurinic and apyrimidinic site formation [19,20,23]. In addition, the spontaneous hydrolytic deamination of cytosine bases can alter DNA, resulting in a non-native uracil base [19,20]. Replication stress represents another form of spontaneous endogenous DNA damage which occurs during the S phase and causes the stalling of replication forks [24]. The intra-S phase checkpoint is responsible for slowing replication forks to allow DNA damage to be repaired and to prevent genetically aberrant cells from progressing to the next phase of the cell cycle [25]. Furthermore, a complex interaction between checkpoint kinase 1 (Chk1), Claspin, and the Timeless (Tim)-Tim-interacting protein (Tipin) complex mediates the intra-S phase checkpoint [26,27].

## 6. Exogenous Deoxyribonucleic Acid Damage

Exogenous DNA damage, originating from external environmental processes, includes ionizing and solar ultraviolet radiation [19,20]. Ionizing radiation generates a wide variety of DNA lesions [19,20]. These include single and dsDNA breaks as well as oxidative modifications of nucleobases and deoxyribose moieties [19,20]. Solar ultraviolet radiation forms cyclobutane pyrimidine dimers which are strongly linked to the aetiology of skin cancer [19,20,23]. Exogenous DNA damage also includes environmental pollutants present in air, water, and food [20]. Harmful chemicals such as second-hand smoke, pesticides (e.g., organophosphates), and toxic metals (e.g., mercury) are metabolised into highly reactive metabolites that chemically react with nitrogenous bases [20]. Ultimately, these chemicals lead to deleterious DNA strand breaks and DNA adducts [20].

## 7. Deoxyribonucleic Acid Damage Response Pathway

The DDR is an integrated signaling and genomic maintenance network which enables cells to withstand threats posed by DNA damage [28,29,30]. The DDR is involved in signaling the presence of DNA damage to DNA repair machinery [28,29,30]. Sensor proteins recognize DNA lesions and prevent replication fork stalling by mediating the amplification of signaling pathways and stimulating transducers and effectors to impact various cellular processes [31]. These cellular processes include stabilizing replisomes (protein complexes responsible for DNA replication), regulating transcription, monitoring the cell cycle, providing energy through autophagy, remodeling chromatin, repairing damaged DNA, processing ribonucleic acid (RNA), and inducing apoptosis [31]. 

**Figure 2:**
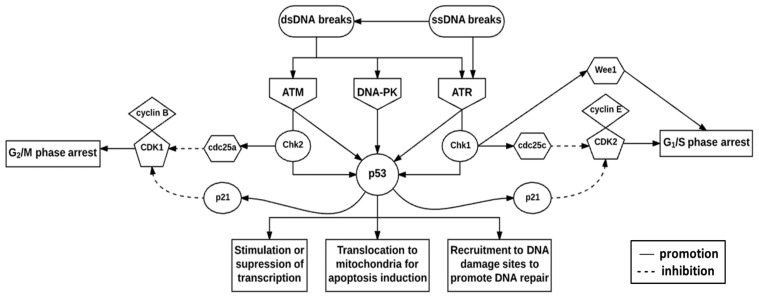
Deoxyribonucleic acid damage checkpoint complex. DNA damage presenting as double-strand DNA (dsDNA)- or single-strand DNA (ssDNA) breaks initiate the DNA damage response (DDR) via ataxia telangiectasia mutated (ATM), DNA-dependent protein kinase (DNA-PK) and ataxia telangiectasia and rad3-related protein (ATR). Checkpoint kinase 2 (Chk2) is expressed throughout the cell cycle and is activated by ATM, whereas checkpoint kinase 1 (Chk1) expression is restricted to the G_1_- and S phases and is activated by ATR. These checkpoint kinases phosphorylate and subsequently activate p53 which integrates stress signals to determine the fate of the cell.

The DNA damage checkpoint complex is composed of sensors, signal transducers, and effector pathways [32]. The fundamental components are the phosphoinositide 3 kinase-related kinases (PIKKs), namely ataxia telangiectasia mutated (ATM), ataxia telangiectasia, and rad3-related (ATR) and DNA-dependent protein kinase (DNA-PK) (Figure 2) [32,33]. These proteins play vital roles in telomere-length regulation to protect chromosome ends from deterioration and in the prevention of their ends fusing with other chromosomes [31,32]. Additionally, the substrates of these proteins, such as Chk1 and checkpoint kinase 2 (Chk2), mediate cell cycle arrest in the G_1_, S, and G_2_ phases of the cell cycle, thus mediating DNA repair and cell death [31,32].

ATM is an important protein involved in the activation of cell cycle checkpoints [34,35]. ATM is recruited to double-strand DNA (dsDNA) breaks by the dsDNA break repair nuclease MRE11-dsDNA break repair protein RAD50 (RAD50)-nibrin (NBS1) (MRN) complex [36]. Upon recruitment, ATM is activated by autophosphorylation at three serine (Ser) sites namely Ser^367^, Ser^1893^, and Ser^1981^ [37]. In addition, ATM is acetylated at lysine (Lys)^3016^ [37]. As a result, ATM phosphorylates the MRN complex and downstream effector proteins such as Chk1 and -2 to initiate cell cycle checkpoints [38,39]. Cell cycle checkpoints allow for increased time to repair DNA damage before the cells enter either the S phase for DNA replication or the M phase for cell division [10,11].

The ATM protein is activated by dsDNA breaks, whereas the ATR protein responds to single-strand DNA (ssDNA) breaks [32,40]. ATM and ATR activate checkpoint regulator substrates Chk2 and Chk1 respectively [31,41]. These checkpoint regulator substrates are responsible for regulating CDK activity [31,32]. Chk1 activates cdc25c phosphatase by phosphorylation which subsequently inhibits CDK2 activity [41,42]. Inhibition of the cyclin E-CDK2 complex results in G_1_/S phase arrest [41]. Chk2 activates cdc25a phosphatase by phosphorylation which subsequently inhibits CDK1 activity [41,42]. Inhibition of the cyclin B-CDK1 complex results in G_2_/M phase arrest [41]. Chk1 also phosphorylates and activates Wee1 kinase to inhibit the G_2_/M phase transition [41,42]. ATM, ATR, DNA-PK, Chk1, and Chk2 are all capable of phosphorylating p53 which regulates the transcriptional activation of p21 to contribute to CDK1- and CDK2-mediated inhibition [40,41]. p53 phosphoprotein mediates various cellular responses to DNA damage, including the regulation of transcription, induction of cell death, and promotion of DNA repair, and is stabilized by post-translational modifications (Figure 2) [31,32]. Cell cycle arrest is promoted for the transcriptional- or post-transcriptional activation of DNA repair proteins [31,32].

## 8. Preservation of Genome Integrity

**Figure 3:**
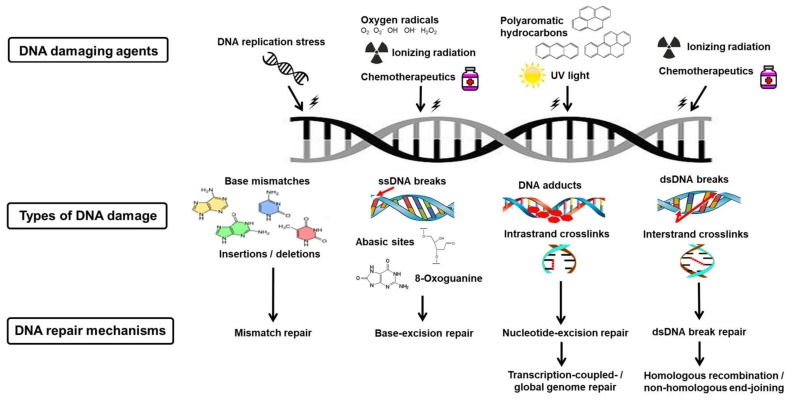
Deoxyribonucleic acid damage and repair mechanisms. Various DNA damaging agents cause a range of DNA lesions. Each are corrected by a specific DNA repair mechanism, namely mismatch repair, base-excision repair, transcription-coupled/global genome repair, or homologous recombination (HR)/non-homologous end-joining (NHEJ).

Cell cycle checkpoint prolongation and DDR protein recruitment is highly dependent on the characteristics and complexity of the DNA damage sustained [2,43]. Therefore, specific DNA repair mechanisms and DNA repair genes are responsible for correcting particular types of DNA lesions [2,43]. The prominent DNA repair mechanisms include mismatch repair, base-excision repair, nucleotide-excision repair, and dsDNA break repair [43,44]. dsDNA break repair can be further divided into three subtypes, namely non-homologous end-joining (NHEJ), homologous recombination (HR), and microhomology-mediated end joining (MMEJ) (Figure 3) [43,44].

## 9. Mismatch Repair

Mismatch repair is responsible for correcting base pair mismatches which occur when adenosine-guanine and cytosine-thymidine do not pair correctly [45]. The specific pathway that detects and removes misincorporated bases was discovered by Paul Modrich (Nobel Prize in Chemistry, 2015) [45]. Mismatch repair also corrects DNA insertions and deletions resulting from erroneous DNA replication or DNA polymerase misincorporation errors (Figure 3) [44,46,47]. Mismatch repair involves three sequential processes: recognition of the mismatch, excision of the incorrect DNA sequence, and resynthesis of the correct DNA sequence [48].

In initiating mismatch repair, the Mutator S (MutS) complex is responsible for detecting DNA mismatches [48,49,50]. The excision of the ssDNA mismatch lesion occurs upon detection of base–base mismatches and insertion/deletion loops (dsDNA with one or more unpaired nucleotides) [31,43,51]. Nuclease, polymerase, and ligase enzymes act on the subsequent ssDNA excision to ensure the new DNA strand is inserted correctly [31]. As mismatch repair is an immediate post-replicative correction mechanism, proteins involved in the repair process are regulated by the cell cycle [43,52]. These DNA mismatch repair proteins include MutS homolog 1 (MSH1) and Mutator L (MutL) homolog 1 (MLH1) which recognise base-base mismatches as well as insertion/deletion loops [43,52].

In the recognition step, MutS is recruited ahead of proliferating cell nuclear antigen (PCNA), an essential DNA replication accessory protein [53]. MutSα (MutS homolog 2 (MSH2)-MutS homolog 6 (MSH6) heteroduplex) recognizes base-base mismatches whereas MutSβ (MSH2-MutS homolog 3 (MSH3) heteroduplex) recognizes insertion/deletion loops [48,49,50,54]. Upon MutS recruitment, the DNA mismatch repair protein MutL is recruited to MutS [48,49,50]. In the excision step, MutL activates Mutator H (MutH) endonuclease to generate a ssDNA break (DNA nick) containing the mismatch which allows for the attachment of exonuclease 1 (Exo1) [48,49,50]. Exo1 excises the mismatched DNA strand. This is proceeded by the recruitment of replication protein A (RPA) to protect the resulting ssDNA [48,49,50]. In the resynthesis step, DNA polymerase synthesizes a new complementary DNA strand to correct the mismatch and DNA ligase repairs the DNA nick and restores the DNA double helix [48,49,50].

## 10. Base-Excision Repair

Base-excision repair is involved in the removal and replacement of damaged DNA bases [55]. Tomas Lindahl described the pathway in which these modified bases are repaired, for which he was awarded the Nobel Prize in Chemistry (2015) [55]. Glycosylases are responsible for the detection and removal of the damaged DNA base(s) and the subsequent forming of an abasic site [56]. DNA damage-causing agents that specifically induce the base-excision repair pathway include ROS (e.g., superoxide (O_2_^−^) and hydrogen peroxide (H_2_O_2_)), X-rays (e.g., computed axial tomography scans), alkylating agents (e.g., cisplatin), and spontaneous reactions (e.g., replication fork stalling) [44,57]. DNA lesions arise from these mutagens via alkylation, deamination, and oxidation reactions, and as a result of ROS-induced oxidative damage to guanine and the subsequent formation of 8-oxoguanine (Figure 3) [43,58].

ROS are generated during normal cellular respiration [23,59]. Electron transfer occurs between various metabolic intermediates and a terminal electron acceptor, namely molecular oxygen (O_2_) during aerobic respiration [23]. ROS have the potential to cause oxidative damage to DNA as a result of their unpaired electrons which make them highly reactive [23]. H_2_O_2_ is a by-product of numerous biochemical reactions such as uric acid formation, but may also be generated by ionizing radiation [23]. H_2_O_2_ produces two oxidized base products, namely 8-oxoguanine, which binds to adenine or cytosine to form a transversion mutation (conversion from a purine to a pyrimidine and vice versa), and thymine glycol which inhibits DNA replication [23,59].

In base-excision repair, a substrate-specific DNA glycosylase enzyme detects a damaged DNA base and removes it by cleaving the *N*-glycosidic bond between deoxyribose and the damaged base [43,56,58,60,61,62]. Nuclease, polymerase, and ligase enzymes are subsequently recruited in order to complete DNA repair in a similar manner as in ssDNA break repair [56,60,61,62]. Upon recognition and excision of a damaged DNA base by a substrate-specific DNA glycosylase enzyme, an abasic site is formed and is cleaved by the apurinic-apyrimidinic endonuclease [56,58,60,61,62]. Scaffolding proteins, namely poly (adenosine diphosphate (ADP)-ribose) polymerase 1 (PARP1) and X-ray repair cross-complementation protein 1 (XRCC1), protect the resulting ssDNA and recruit downstream base-excision repair proteins [56,60,61,62]. In short-patch base excision repair, DNA polymerase β inserts the modified base and DNA ligase I or -III seal the remaining DNA nick [56,58,60,61,62]. In long-patch base excision repair, DNA polymerases δ and -ε insert the correct bases past the gap, while flap endonuclease 1 (FEN1) cleaves the displaced DNA and DNA ligase 1 along with PCNA which seals the nick [56,58,60,61,62]. 

Base excision repair corrects large numbers of small DNA base lesions caused by alkylation, deamination, and oxidation reactions [43,44]. Components of base excision repair machinery such as glycosylases are regulated in a cell cycle-specific manner [43]. The expression of uracil-DNA glycosylase, encoded by the UNG gene, peaks in the late G_1_ phase continuing throughout the S phase [43,58]. The expression of thymine/uracil mismatch glycosylase, encoded by the TDG gene, peaks in the G_1_ phase and declines in the S phase [43,58].

## 11. Nucleotide-Excision Repair

Nucleotide-excision repair controls the removal of DNA adducts (segments of DNA covalently bound to carcinogenic chemicals) from DNA by excising an oligonucleotide containing the lesion to replace it with newly synthesised DNA [63]. The discovery of the mechanism by which this is achieved is attributed to Aziz Sancar (Nobel Prize in Chemistry, 2015) [63]. DNA-damaging agents that induce the nucleotide-excision repair pathway include ultraviolet light and polycyclic aromatic hydrocarbons which contribute to destabilization of the DNA double helix [44,64]. These agents may cause DNA adducts, such as etheno-DNA adducts (e.g., 1,*N*^6^-ethenodeoxyadenosine and 3,*N*^4^-ethenodeoxycytidine), which are generated from exogenous carcinogen metabolism and endogenous lipid peroxidation, as well as intrastrand crosslinks, characterised by the covalent binding of nucleotides within the same DNA strand (Figure 3) [43,65,66].

DNA double helix-distorting lesions are recognized by the xeroderma pigmentosum group A (XPA) protein and undergo repair in one of two pathways depending on the type of lesion i.e., transcription-coupled nucleotide-excision repair or global genome nucleotide excision repair [31,67]. DNA double helix distortion is most commonly caused by pyrimidine dimers formed by ultraviolet light [43,44]. Transcription-coupled nucleotide excision repair targets lesions blocking transcription while global genome nucleotide-excision repair targets lesions in both transcribed- and non-transcribed DNA [31,67]. Nucleotide excision repair is characterised by the excision of the 25–30 base oligonucleotide segments containing the adduct, resulting in ssDNA on which DNA polymerases act before ligation occurs [68].

Global genome nucleotide excision repair is initiated by the xeroderma pigmentosum group C-RAD23 homolog B (XPC-RAD23B) complex which binds to the non-damaged DNA strand opposite to the lesion [68,69,70]. Transcription factor II human (TFIIH) interacts with XPC-RAD23B to recruit the group B subunit (XPB) to separate DNA strands and allow the group D subunit (XPD) to detect DNA damage and verify the chemical composition of the lesion [68,69,70]. The pre-incision complex is formed with the recruitment of RPA, the group A subunit (XPA) and the group G subunit (XPG) [68,69,70]. The excision repair cross-complementation group 1-xeroderma pigmentosum group F (ERCC1-XPF) complex interacts with XPA to form a 5′ DNA incision at the lesion [68,69,70]. DNA repair synthesis is initiated by polymerases δ and -κ or polymerase ε and is followed by a 3′ DNA incision at the lesion by XPG [68,69,70]. The DNA nick is sealed by DNA ligase I or the DNA ligase IIIa-XRCC1 complex [68,69,70].

## 12. Double-Strand Deoxyribonucleic Acid Break Repair

DsDNA breaks occur when the sugar-phosphate backbones of both DNA strands are broken at a similar position or in close proximity to one other [71]. Subsequently, physical dissociation of the DNA double helix takes place resulting in the formation of two separate single-stranded molecules [71]. Genetic information is lost as a result of the absence of a DNA template for accurate repair in the newly synthesized DNA [71]. DNA-damaging agents that cause dsDNA breaks and the repair pathway include X-rays, ionizing radiation, and anti-cancer drugs (e.g., cisplatin) [44,72]. These agents may also cause other DNA lesions such as interstrand crosslinks (covalent bonds which form between complementary strands thereby inhibiting their separation and replication) (Figure 3) [43]. Replication fork stalling may be the result of dsDNA-damaging agents or may be responsible for the formation of dsDNA breaks as a result of origin re-firing in an attempt to promote replication fork speed [25,73]. Thus, dsDNA break repair mechanisms are essential for replication fork progression and stable DNA replication [25,73].

The MRN complex detects dsDNA breaks and subsequently recruits and activates members of the DDR machinery such as those of the phosphatidylinositol 3 kinase (PI3K) family [74,75]. Activated ATM phosphorylates histone variant H2AX at Ser^139^ resulting in the formation of foci at sites of DNA damage lending to the recruitment of repair proteins [76,77]. Although the phosphorylated form of histone H2AX (γ-H2AX) may be regarded as a sensitive quantitative indicator of dsDNA damage, specificity is not high as it may also serve as evidence of other DNA stressors such as stalled replication forks [78].

With dsDNA breaks, two principle mechanisms may be implemented in the repair process, namely NHEJ and HR [79]. This classification depends on whether sequence homology (template DNA sequence) is used to join dsDNA break ends [79,80]. In NHEJ, sequence homology is not required for dsDNA break end-joining and it involves minimal DNA processing [79,80]. In HR, sequence homology is required in order to align dsDNA break ends prior to ligation [79,80].

**Figure 4:**
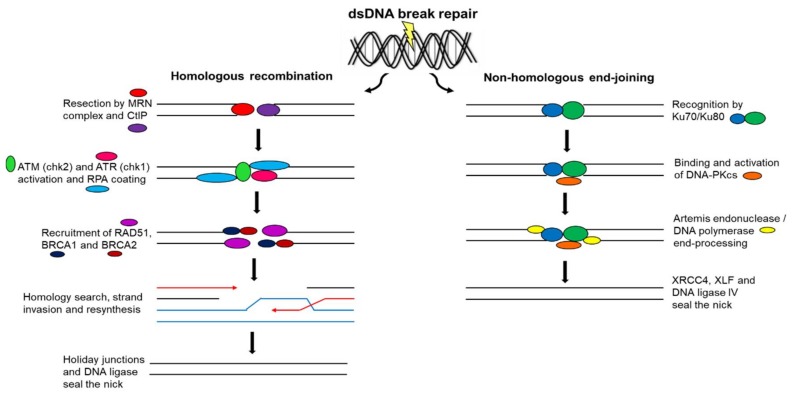
Double-strand deoxyribonucleic acid break repair. DsDNA breaks are repaired by HR or NHEJ. HR involves the restoration of DNA sequences using sister chromatid sequence homology as a template and functions in all phases of the cell cycle, whereas NHEJ involves damaged DNA sequence deletions and functions in only S and G_2_ phases.

The Ku70/Ku80 heterodimer recognizes dsDNA breaks by binding to both blunt or near-blunt broken DNA ends to elicit NHEJ [81,82,83]. Additionally, it binds and activates the DNA-PK catalytic subunit (DNA-PK_CS_) [81,82]. NHEJ is facilitated by scaffold proteins, namely X-ray repair cross-complementation protein 4 (XRCC4) and XRCC4-like factor (XLF), which bind to DNA ligase IV to seal the DNA nick [81,82,83]. DNA end-processing occurs prior to ligation to ensure compatible DNA ends by either the DNA-PK_CS_-interacting protein Artemis endonuclease trimming DNA ends or polymerases filling DNA ends (Figure 4) [81,82,84]. MMEJ, also known as alternative end-joining, is a Ku protein-independent NHEJ pathway that commonly results in DNA sequence deletions [71]. Both NHEJ and MMEJ can function in all phases of the cell cycle [71].

A paralog of XRCC4 and XLF (PAXX), a member of the XRCC4 superfamily, is recruited to dsDNA damage breaks and interacts directly with the Ku70/Ku80 heterodimer to initiate NHEJ [85,86]. Moreover, PAXX functions with structurally similar scaffold proteins XRCC4 and XLF to facilitate ligation of the DNA nick and conclude dsDNA break repair [85,86]. PAXX is a novel component of the NHEJ machinery and promotes cell survival in response to dsDNA break-inducing agents [85,86].

In HR, replicated sister chromatid DNA sequences are used as templates to restore missing DNA sequences on the damaged chromatid [87,88]. For this reason, HR can only operate in the S and G_2_ phases of the cell cycle when replicated sister chromatids are available [87,88]. HR is initiated by resection of broken DNA ends by the MRN complex and the C-terminal-binding protein interacting protein (CtIP) generating 3′-ssDNA tails [87,88,89]. RPA coats 3′-ssDNA tails and is replaced by RAD51 with the help of breast cancer gene 1 (*BRCA1*) and 2 (*BRCA2*) [79,87,88,89]. A nucleoprotein complex is created that detects the homologous sequence on the sister chromatid, a process referred to as strand invasion [89,90]. RAD51 catalyzes strand invasion on the homologous template to allow the restoration of lost sequence information [87,90]. Broken DNA ends are resolved by junctions called Holiday junctions that result in crossover and non-crossover products (Figure 4) [84]. As HR makes use of a non-damaged DNA template to restore chromosome integrity, it is a more precise method of DNA repair than that of NHEJ and MMEJ [71].

Two additional dsDNA break repair pathways exist that require sequence homology, namely single-strand annealing (SSA) and alternative non-homologous end-joining (alt-NHEJ) [91]. SSA is mediated by a single-strand annealing protein (SSAP) (such as RAD52) which uses a short single-stranded region to locate sequence identity and initiate HR [91,92]. In the SSA pathway, resection of the dsDNA break exposes complementary sequences in the ssDNA tails of the two ends [93]. Complementary sequences anneal, leaving non-complementary flaps of ssDNA [93]. Nucleolytic flap removal and ligation finalizes the repair of the dsDNA break [93]. In the absence of XRCC4 and DNA ligase IV, which are responsible for concluding the classical NHEJ repair pathway, alt-NHEJ resolves the dsDNA break [94]. Alt-NHEJ relies on CtIP-dependent resection as in HR but requires a unique set of repair factors, namely PARP1 together with DNA ligase I or III [95,96].

Aurora kinase A and ninein-interacting protein (AUNIP) acts as a dsDNA damage sensor and interacts with CtIP to ensure adequate accumulation of CtIP at dsDNA breaks to initiate DNA end resection and subsequently HR [97,98]. AUNIP is recruited to dsDNA damage sites through a DNA-binding motif displaying a preference for substrates that are structurally similar to those formed at replication forks during replication stalling [97]. The absence of AUNIP results in the failure of the HR pathway and is accompanied by hypersensitivity to DNA damage agents that cause replication-associated dsDNA breaks [97]. When AUNIP is overexpressed, it promotes HR and inhibits NHEJ; however, when AUNIP is inhibited, the frequency of NHEJ is increased [97].

Ubiquitin-specific protease 4 (USP4) facilitates HR by directly participating in dsDNA break end-resection through the post-translational process of autoubiquitination [99,100]. USP4 interacts with both CtIP and the MRN complex via a specific conserved region on USP4 and the catalytic domain of USP4, respectively, to promote the recruitment of the DNA end-resection factor CtIP to dsDNA break sites [100]. USP4 contains several ubiquitinated sites, mostly on cysteine residues [99]. USP4 catalytic activity is responsible for the deubiquitination of these cysteine residues to promote CtIP recruitment [99]. USP4 is a novel HR regulator whose enzymatic activity is regulated by ubiquitin adducts [99,100].

## 13. Pathophysiology of Deoxyribonucleic Acid Repair Failure

Rare hereditary diseases characterized by DNA repair deficiencies arise when repair machinery and mechanisms are defective [101]. Germline mutations in the relevant DNA repair genes are responsible for a range of diseases including Werner, Bloom, and Cockayne syndromes [101].

Mismatch repair deficiencies are present in adult-onset autosomal dominant Lynch syndrome and child-onset autosomal recessive constitutional mismatch repair deficiency syndrome, which include well-established colorectal and endometrial cancer syndromes [31,101,102]. Both adult- and child-onset hereditary tumour syndromes are characterized by mutated MSH and MLH genes which are responsible for sensing base-base mismatches and insertion/deletion loops (Table 1) [31,101,102]. Xeroderma pigmentosum is a non-curable genetic disease characterized by germline mutations in nucleotide-excision repair genes causing neurodegeneration, photosensitivity, and skin cancer [31,101,103]. Mutated xeroderma pigmentosum genes foster the creation of altered protein products which are responsible for the inability to repair DNA adducts and intrastrand crosslinks resulting from defective recognition and signaling of these nucleotide lesions (Table 1) [103]. Spinocerebellar ataxia with axonal neuropathy (SCAN1) results from a tyrosyl-DNA phosphodiesterase deficiency, an enzyme involved in controlling DNA winding by topoisomerase during base excision repair [104]. Ataxia with oculomotor apraxia 1 (AOA1) results from an aprataxin deficiency which is associated with scaffolding proteins which facilitate accurate base-excision repair [105]. Both SCAN1 and AOA1 are characterized by base excision repair deficiencies that produce ataxia and neurodegeneration (Table 1) [104,105]. DDR defects are present in Li-Fraumeni syndrome in which soft tissue sarcomas, breast cancer, and brain tumours are prevalent [31,101,106]. A mutation in the p53 gene inhibits the DDR and interferes with cell cycle regulation and tumour suppression (Table 1) [106]. If any component of the DDR machinery is impaired, it results in defective DNA damage sensing and signaling, potentially leading to pathological conditions that include Alzheimer’s, Parkinson’s, and Huntington’s diseases [31,101].

The identification of various pathologies related to inadequate DNA damage detection and DNA repair mechanisms highlights the fundamental role these processes play in maintaining genomic stability [107]. These networks are of particular importance in the prevention of neurodegeneration and malignant transformation [107]. Furthermore, they govern normal growth, neurogenesis, and immune system development [107].

## 14. Deoxyribonucleic Acid Repair Pathways as Therapeutic Targets and Future Directions

A defective DDR system is a hallmark of certain cancers which allows tumour cells to proliferate and acquire mutations [108]. These DNA repair defects serve as a platform for the discovery of specific treatments for selected cancers [108].

PARP1 binds to damaged DNA ends via two homologous N-terminal zinc (Zn) finger domains, Zn1 and Zn2 [109]. The carboxylic catalytic domain of PARP1 uses nicotinamide adenine dinucleotide (NAD^+^) as a substrate to synthesize poly (ADP-ribose) chains by an autoregulated process [109]. A structurally unique third Zn finger domain, Zn3, plays a vital role in the synthesis of these poly (ADP-ribose) chains [110]. Poly (ADP-ribose) chain synthesis at the carboxylic catalytic domain leads to the recruitment of the base-excision repair complex through interaction with condensin I and XRCC1 [110,111]. When PARP1 is bound to damaged DNA ends it prevents the conversion of ssDNA breaks into dsDNA breaks until base excision repair is completed [110].

Defects in HR proteins, specifically *BRCA1* and -*2*, lead to the failure of dsDNA break repair and increase the likelihood of breast and ovarian cancer [112,113]. Cancer cells harbouring *BRCA* mutations are unable to recruit RAD51 to dsDNA break sites during HR, thus forcing cells into the more error-prone NHEJ repair pathway [114]. This HR defect promotes tumour cell sensitivity to treatments that induce ssDNA breaks [115]. One such treatment strategy is the inhibition of scaffold protein PARP1 which is involved in the repair of ssDNA lesions [114,116,117]. Furthermore, PARP inhibition leads to an accumulation of dsDNA aberrations giving rise to cell death, a process referred to as synthetic lethality [114,116,117].

ATM regulates responses associated with dsDNA break repair by phosphorylating downstream regulatory proteins and repair factors such as *BRCA1*, Chk2 and p53 [118]. Williamson et al. (2012) showed that mantle cell lymphoma expressing ATM and p53 mutations exhibit enhanced cytotoxicity to olaparib (PARP inhibitor) treatment both in vitro and in vivo [119]. In addition, intact DNA-PK, together with mutated ATM/p53, contribute to the induction of NHEJ as well as the synthetic lethal response consequent to PARP inhibition [119]. PARP activity is required for the detection and resumption of stalled replication forks following replication stress [120]. Following recognition by PARP, the MRN complex is recruited and the HR repair pathway repairs the damage in order to restart the replication fork [121,122]. PARP inhibition thus prevents the downstream processes required for the continuation of replication forks and subsequent DNA replication [122]. Cytogenetic aberrations involving chromosome 11q, which contains cancer-associated genes such as ATM and Chk1, have been implicated in neuroblastoma [123]. Defective DDR systems display a sensitivity to PARP inhibition, and thus PARP inhibitors are promising neuroblastoma therapeutics [123].

Olaparib was approved in 2014 by the Food and Drug Administration (FDA) as a monotherapy for women diagnosed with *BRCA*-deficient or -mutant ovarian cancer who had undergone three or more failed chemotherapy regimens [124]. The administration of olaparib within this patient subset resulted in progression-free survival that was significantly longer in the olaparib treatment group (48%) when compared to the placebo group (15%) [125]. Olaparib has a good oral bioavailability but myelodysplastic syndrome and acute myeloid leukaemia have been reported as more substantive unwanted effects [124,126]. Olaparib is the first clinical chemotherapeutic agent inhibiting PARP in order to target DNA repair defects in malignant cells [127].

DNA strand break bait (Dbait) molecules are DNA repair inhibitors that mimic dsDNA breaks and sequester dsDNA break repair proteins such as DNA-PK and PARP1 [128]. These large molecules are comprised of 32-base pair double helices that interfere with dsDNA break signaling by acting as bait for repair enzymes and thus inhibit HR and NHEJ [128]. Dbait molecules cause DNA-PK hyper-activation, resulting in the phosphorylation of DNA damage signaling molecules, including H2AX, Chk2, and p53, ultimately preventing the recruitment of DNA repair complexes to DNA damage sites [129].

Biau et al. (2014) conducted a preclinical study in which a cholesterol-conjugated Dbait molecule, DT01, sensitized melanoma cells to radiotherapy both in vitro and in vivo [128]. In addition, DT01 has been shown to improve the efficacy of the chemotherapeutic doxorubicin in mouse models bearing hepatocellular carcinoma [130]. Herath et al. (2016) investigated the chemosensitizing effects of DT01 in combination with a two-drug chemotherapeutic regimen (oxaliplatin and 5-fluorouracil) in an in vivo colorectal liver metastases model, and have reported significant anti-tumour effects using the combined treatment [131]. Moreover, H2AX phosphorylation by DNA-PK was exclusive to tumour cells, thus indicating sparing of surrounding non-tumourigenic tissue [131]. A signal-interfering DNA (AsiDNA), which is a cholesterol-conjugated member of the Dbait family, induces preferential toxicity towards tumourigenic tissue whilst sparing non-tumourigenic hematologic cells and preserving immune function [132]. Thierry et al. (2017) reported the induction of necrotic and apoptotic cell death by AsiDNA through p53-independent mechanisms in several lymphoma and leukaemia cell lines [132]. AsiDNA enters cells through low density lipoprotein (LDL) receptors and subsequently activates DNA-PK [132]. Dbait molecules improve the clinical outcomes of chemo- and radiotherapy by disturbing DNA repair processes in treated tumour tissue [128,132,133]. The combination of PARP inhibitor and Dbait leads to increased unrepaired dsDNA breaks, resulting in amplified tumour cell death while sparing non-tumour cells [133].

PARP inhibitors constitute a major emerging class of promising therapeutics; however, various other DNA repair pathway inhibitors are also currently being investigated [134]. Preclinical and clinical development of highly selective small molecule inhibitors of ATM and ATR is aimed at targeting the DDR and subsequently DNA repair [135,136,137]. Base excision repair inhibitors include apurinic-apyrimidinic endonuclease inhibitors that prevent abasic site cleavage and DNA polymerase β inhibitors, preventing the insertion of modified bases [134,138]. Protein-protein and protein-DNA interactions involved in nucleotide-excision repair have been identified as targets of the repair pathway and include ERCC1-XPF, ERCC1-XPA, and RPA-DNA [134,139]. Inhibition of vital proteins involved in NHEJ, such as DNA-PK and the Ku70/Ku80 heterodimer, inhibit recognition of termini and end-bridging, thus implicating dsDNA break repair [140,141]. Furthermore, inhibition of vital components of HR machinery such as RAD51 targets the alternative dsDNA break repair pathway [142].

Genetic engineering is an emergent experimental treatment strategy with potential applications in incurable genetic disorders [143]. One area which has received considerable attention is gene editing and, in particular, the use of the clustered regularly interspaced short palindromic repeats (CRISPR)/CRISPR-associated nuclease (Cas) system [143]. There are three CRISPR/Cas systems which are classified according to a specific Cas protein [143]. Type I is identifiable by the presence of Cas3, type II by Cas9, and type III by Cas10 [143]. Each system uses a unique mechanism to recognize and cleave nucleic acids [144]. Type I and III use Cas complexes to target specific DNA sites; however, type II requires only a single Cas protein [144]. For this reason, the type II CRISPR/Cas9 system has been used preferentially for genetic engineering [144,145]. CRISPR/Cas9 uses a complementary guide-RNA (gRNA) sequence and a protospacer adjacent motif (PAM) sequence to recognize a specific targeted DNA sequence [144,145]. PAM sequences, usually 5′-asparagine-glycine-glycine-3′(5′-NGG-3′), are used by both type I and -II CRISPR/Cas systems to recognize target DNA [146]. PAM sequences lie within the target DNA sequence and, if absent, Cas9-binding will not occur even if the gRNA sequence is complementary to the target DNA sequence [147]. The C-terminal of Cas9 interacts with the PAM sequence via arginine (Arg) motifs 1333 and -1335 to trigger the separation of the upstream strands of the target sequence at the first base pair position [148]. Cas9 has two catalytic nuclease domains, histidine-asparagine-histidine (HNH) and RuvC, each of which are responsible for cleaving one DNA strand in a coordinated manner [149,150]. The HNH domain cleaves the complementary strand while the non-complementary strand is cleaved by the RuvC domain [149,150]. This concurrent cleaving activity produces a dsDNA break which is repairable by either HR or NHEJ [150,151,152]. Error-prone repair pathways such as NHEJ may result in gene-silencing or insertion/deletion mutations which are generally used for gene knock-out experiments [150,151,152]. The homology-directed repair pathway ensures precise DNA repair through a ‘copy-paste’ mechanism using a donor template; however, NHEJ is the preferred repair pathway in response to Cas9 cleavage [151,153]. 

Although the potential clinical applications of CRISPR/Cas9 are numerous, these are in early stages of research [154]. CRISPR/Cas9 may be applicable in the treatment of cancer and genetic disorders such as Duchenne muscular dystrophy, retinitis pigmentosa, β-thalassaemia, and xeroderma pigmentosum disorder [154,155,156]. Huntington’s disease is characterized by an expansion of cytosine-adenine-guanine/glutamine repeats; a potential therapeutic target may lie in the use of CRISPR/Cas9 to silence the mutant form of the huntingtin gene (mHtt) [152].Yang et al. (2017) demonstrated successful CRISPR/Cas9 suppression of mHtt in mouse striatal neuronal cells which resulted in the alleviation of motor impairments and neurotoxicity [152]. Ou et al. (2016) combined CRISPR/Cas9 technology with that of Takahashi and Yamanaka’s (2006) induced pluripotent stem cells (iPSCs) with the aim of curing β-thalassaemia by correcting the defective β-globin gene [157,158]. Corrected iPSCs were used to generate haematopoietic stem cells that could successfully differentiate and survive in mice without exhibiting tumourigenic properties [158]. CRISPR/Cas9 is a promising tool harnessing DNA damage as well as repair pathways and mechanisms in the treatment of incurable diseases, disease mapping, drug screening, and personalized medicine [144,145].

## 15. Conclusions

DNA is responsible for carrying hereditary information across generations; it accomplishes this by controlling the production and function of proteins. As a result, it is essential for growth, survival, and reproduction. Should aberrations occur in DNA, genome integrity is maintained through accurate DNA replication and adequate DNA repair. DNA damaging agents may be of endogenous or exogenous origin and the resulting DNA lesions may cause morbidity and mortality if not repaired. Specific DNA repair mechanisms that are closely associated with the cell cycle exist to correct the different types of DNA lesion that occur. Failure of these vital repair processes may lead to a variety of mutations and, consequently, diseases. Through an understanding of the causes of DNA damage and the corresponding repair mechanisms, it is possible to design strategies to create or improve methods for the prevention, diagnosis, and treatment of pathologies related to deficiencies in the mechanisms of DNA repair.

## Figures and Tables

**Table 1:** Diseases and disorders associated with defective DNA repair.

DNA Repair Mechanism	Associated Disease/Disorder	Mutation/Deficiency Responsible	Clinical Presentation
Mismatch repair	Lynch syndrome Constitutional mismatch repair deficiency syndrome	MSH and MLH mutations [103]	Colorectal cancer, endometrial cancer [103]
Nucleotide-excision repair	Xeroderma pigmentosum disorder	Mutations in xeroderma pigmentosum complexes [104]	Neurodegeneration, photosensitivity, skin cancer [104]
Base-excision repair	Spinocerebellar ataxia with axonal neuropathy (SCAN1) Ataxia with oculomotor apraxia 1 (AOA1)	Tyrosyl-DNA phosphodiesterase deficiency [105] Aprataxin deficiency [106]	Ataxia, neurodegeneration [105,106]
DNA damage response (DDR)	Li-Fraumeni syndrome	p53 mutation [107]	Soft tissue sarcomas, breast cancer, brain tumours [107]

## Abbreviations

ADP	adenosine diphosphate
alt-NHEJ	alternative non-homologous end-joining
AOA1	ataxia with oculomotor apraxia 1
Arg	arginine
AsiDNA	a signal interfering DNA
ATM	ataxia telangiectasia mutated
ATR	ataxia telangiectasia and rad3-related
AUNIP	aurora kinase A and ninein interacting protein
*BRCA1*	breast cancer gene 1
*BRCA2*	breast cancer gene 2
CAKs	CDK activating kinases
Cas	CRISPR-associated nuclease
cdc25	cell division cycle 25
CDKs	cyclin-dependent kinases
CDKIs	CDK inhibitors
Chk1	checkpoint kinase 1
Chk2	checkpoint kinase 2
CRISPR	clustered regularly interspaced short palindromic repeats
CtIP	C-terminal-binding protein interacting protein
Dbait	DNA strand break bait
DDR	DNA damage response
DNA	deoxyribonucleic acid
DNA-PK	DNA-dependent protein kinase
DNA-PK_CS_	DNA-PK catalytic subunit
dsDNA	double-strand DNA
ERCC1	excision-repair cross-complementation group 1
Exo1	exonuclease 1
FDA	Food and Drug Administration
FEN1	flap endonuclease 1
G	glycine
G_0_	resting
G_1_	growth 1/gap 1
G_2_	pre-mitotic/gap 2
gRNA	guide RNA
H	histidine
H_2_O_2_	hydrogen peroxide
HR	homologous recombination
INK	CDK4 inhibitor
iPSCs	induced pluripotent stem cells
KIP	CDK inhibitor
LDL	low density lipoprotein
Lys	lysine
M	mitotic
mHtt	mutant huntingtin gene
MLH1	MutL homolog 1
MMEJ	microhomology-mediated end-joining
MRE11	dsDNA break repair nuclease MRE11
MRN	MRE11-RAD50-NBS1
MSH1	MutS homolog 1
MSH2	MutS homolog 2
MSH3	MutS homolog 3
MSH6	MutS homolog 6
mTOR	mammalian target of rapamycin
MutH	Mutator H
MutL	Mutator L
MutS	Mutator S
MutSα	MSH2-MSH6 heteroduplex
MutSβ	MSH2-MSH3 heteroduplex
Myt1	myelin transcription factor 1
N	asparagine
NAD^+^	nicotinamide adenine dinucleotide
NBS1	nibrin
NHEJ	non-homologous end-joining
O_2_	oxygen
O_2_^−^	superoxide
PAM	protospacer adjacent motif
PARP1	poly (ADP-ribose) polymerase 1
PAXX	paralog of XRCC4 and XLF
PCNA	proliferating cell nuclear antigen
PI3K	phosphatidylinositol 3 kinase
PIKKs	phosphoinositide 3 kinase-related kinases
RAD50	dsDNA break repair protein RAD50
RNA	ribonucleic acid
RNS	reactive nitrogen species
ROS	reactive oxygen species
RPA	replication protein A
S	DNA synthesis
SCAN1	spinocerebellar ataxia with axonal neuropathy
Ser	serine
SSA	single-strand annealing
SSAP	single-strand annealing protein
ssDNA	single-strand DNA
TFIIH	transcription factor II human
Tim	Timeless
Tipin	Tim-interacting protein
USP4	ubiquitin-specific protease 4
XLF	XRCC4-like factor
XPA	xeroderma pigmentosum group A
XPB	xeroderma pigmentosum group B
XPC-RAD23B	xeroderma pigmentosum group C-RAD23 homolog B
XPD	xeroderma pigmentosum group D
XPF	xeroderma pigmentosum group F
XPG	xeroderma pigmentosum group G
XRCC1	X-ray repair cross-complementation protein 1
XRCC4	X-ray repair cross-complementation protein 4
Zn	zinc
